# Global burden of polycystic ovary syndrome among women of childbearing age, 1990–2021: a systematic analysis using the global burden of disease study 2021

**DOI:** 10.3389/fpubh.2025.1514250

**Published:** 2025-03-26

**Authors:** Yaping Meng, Ting Zhao, Rui Zhang, Xiaoyan Zhu, Chao Ma, Qin Shi

**Affiliations:** ^1^Department of Obstetrics and Gynecology, Jiading District Central Hospital Affiliated Shanghai University of Medicine and Health Sciences, Shanghai, China; ^2^School of Clinical Medicine, Shandong Second Medical University, Weifang, China

**Keywords:** polycystic ovary syndrome, women of childbearing age, global burden of disease study, socio-demographic index, epidemiological trends

## Abstract

**Background:**

The escalating global incidence of polycystic ovary syndrome (PCOS) necessitates a thorough examination of its epidemiological trends and sociodemographic correlations. Our study bridges this gap by analyzing the global impact of PCOS among women of childbearing age (WCBA) from 1990 to 2021, aiming to inform strategies for enhanced prevention and management.

**Methods:**

We extracted data from the Global Burden of Disease Study 2021 (GBD 2021), focusing on the burden of PCOS among women aged 15–49 years. We assessed incidence, prevalence, and disability-adjusted life years (DALYs) trends using the estimated annual percentage change (EAPC) and explored the link between PCOS burden and sociodemographic index (SDI).

**Results:**

In 2021, the prevalence cases of PCOS worldwide were 65.77 million, the incidence cases were 1,175.07 thousand, and the DALYs cases were 576.05 thousand. Compared with 1990, the percentage changes were 89, 49, and 87%, respectively. The EAPCs indicated upward trends in prevalence and DALYs rates, with a less pronounced increase in incidence rates. The middle SDI region had the highest PCOS case numbers, and the 45–49 age group in this region experienced the most significant burden increase. A strong positive correlation existed between PCOS prevalent rates and SDI (*r* = 0.582, *p* < 0.001).

**Conclusion:**

The global burden of PCOS among WCBA has significantly increased over the past three decades, particularly in the 45–49 age group in middle SDI regions. The correlation between PCOS prevalent rates and SDI highlights the role of socio-economic factors in PCOS epidemiology. Tailored prevention and management strategies are crucial for reducing the global burden of PCOS and improving female health outcomes.

## Introduction

Polycystic ovary syndrome (PCOS) is a widespread endocrine and metabolic disorder affecting women of childbearing age (WCBA), with a global prevalence that is estimated to be between 5 and 21% (1–3). This condition is characterized by chronic anovulation, elevated androgen levels, and the presence of multiple ovarian cysts (4, 5). Clinically, PCOS presents with a variety of symptoms such as menstrual irregularities, infertility, excessive hair growth, acne, obesity, and insulin resistance, which contribute to it being a major cause of reproductive and metabolic issues in women (6, 7). Moreover, PCOS is linked to significant long-term health risks. Women with PCOS, especially those with hyperandrogenic traits, are approximately 2.5 times more likely to develop type 2 diabetes and have a higher risk of cardiovascular diseases compared to the general female population (8–10). Additionally, PCOS patients exhibit elevated prevalence of mental health disorders including depression, anxiety, and eating disorders, which amplify disease impact and compromise holistic quality of life (11).

PCOS not only affects immediate reproductive health but also has enduring implications for long-term health. While hyperandrogenic symptoms may lessen with age, leading to a decrease in clinical manifestations post-menopause, the metabolic and cardiovascular complications associated with PCOS continue into later life (12–14). This reduction in overt symptoms complicates diagnosis, particularly in older women who may no longer meet standard diagnostic criteria but continue to experience adverse health outcomes. Therefore, it is essential to address the burden of PCOS during the reproductive years to ensure early detection, prompt intervention, and the prevention of chronic complications. Gaining a deeper understanding of the disease during this critical life stage is vital for reducing its impact on female health and enhancing quality of life.

The Global Burden of Disease Study 2021 (GBD 2021) offers a comprehensive and systematic framework for quantifying the impact of diseases globally, regionally, and nationally (15, 16). By estimating key epidemiological metrics such as incidence, prevalence, and disability-adjusted life years (DALYs), the GBD offers valuable insights into the health effects of various conditions across diverse populations and time frames. Furthermore, the socio-demographic index (SDI), which reflects socio-economic development factors such as income, education, and fertility, is likely to significantly influence the epidemiological profile of PCOS (17–19). Despite the recognized global health significance of PCOS, few studies have systematically analyzed its burden on a global scale, with most existing research being confined to specific regions or countries without considering broader socio-economic and demographic factors.

Leveraging GBD data to assess the global burden of PCOS provides crucial insights into its epidemiology. Firstly, it enables the identification of high-risk regions and populations, facilitating targeted public health strategies and resource allocation to enhance PCOS diagnosis and management. Secondly, analyzing SDI-related trends can elucidate how socio-economic development influences the distribution and clinical outcomes of PCOS, providing a nuanced understanding of disparities in healthcare access, diagnosis, and treatment across different regions. Lastly, longitudinal data on incidence, prevalence, and DALYs provide valuable perspectives on how the burden of PCOS has changed over time, revealing temporal trends that may guide future interventions.

In this study, we utilize GBD 2021 data to conduct a comprehensive assessment of the global burden of PCOS among WCBA from 1990 to 2021. Our analysis examines trends in incidence, prevalence, and DALYs, while also investigating the relationship between these metrics and SDI. The aim of this study is to provide an in-depth understanding of the epidemiological patterns of PCOS, which will aid in the development of evidence-based public health policies and clinical management strategies. Ultimately, our findings will contribute to improving early detection, prevention, and comprehensive management of PCOS, thereby enhancing the health and well-being of women worldwide.

## Methods

### Data source and disease definition

Our analysis is based on data extracted from the Global Burden of Disease Study 2021 (GBD 2021), which offers the most current estimates of the epidemiological burden for over 371 diseases and injuries across 21 regions and 204 countries/territories from 1990 to 2021 (15, 20–22). The dataset, which is publicly accessible, can be found on the Global Health Data Exchange (GHDx). Detailed methodologies concerning data sources and statistical models are thoroughly documented in previous publications.

In the GBD 2021, the reference definition of PCOS is based on the definition proposed by the American College of Obstetricians and Gynecologists (ACOG). According to ACOG, PCOS is an endocrine disorder characterized by hyperandrogenism, ovulatory dysfunction, and polycystic ovarian morphology (23–25). Its diagnosis can be established using any of the three major diagnostic criteria: the National Institutes of Health (NIH) criteria, the Rotterdam criteria, or the AE-PCOS criteria. Moreover, ethical approval is not required as human subjects are not directly involved.

### Socio-demographic index

The SDI, developed by the Institute for Health Metrics and Evaluation (IHME), serves as a composite measure to assess socio-economic development and its influence on health outcomes. It is calculated as the geometric mean of three key variables: fertility rates among women under 25, average educational attainment for individuals aged 15 and older, and income per capita adjusted for lag time (26). SDI values range from 0, indicating the lowest level of socio-economic development, to 1, representing the highest. Countries and regions are categorized into five SDI regions, including low, low-middle, middle, high-middle, and high, based on their SDI scores ^34^. Understanding the relationship between SDI and PCOS is crucial for identifying disparities in disease burden across different socio-economic strata. Our study aims to analyze this association to identify areas with insufficient medical services and guide policy decisions on resource allocation for PCOS prevention and management.

### DALYs

DALYs is an important metric used to comprehensively assess the impact of diseases on healthy life expectancy (20). It combines years of life lost (YLL) due to premature death with years lived with disability (YLD) resulting from health impairments. By accounting for factors such as incidence, mortality, average age of onset, and duration of illness, DALYs provide a holistic view of disease burden. The key parameters for calculating DALYs include age, sex, age of onset, age at death, and disability weight (DW). As a critical tool in GBD studies, DALYs provide researchers and policymakers with key insights into the impact of various diseases on population health.

### Estimated annual percentage change

EAPC is a key statistical measure used to assess the annual trend of a specific indicator over a continuous period, providing a scientific basis for public health policy and resource allocation (27). EAPC is derived through linear regression analysis and offers a more detailed view of time series changes compared to simply comparing the start and end years.

### Data analysis

Data cleaning, calculations, and visualizations were performed using R software (version 4.2.1).

## Results

### Global level

**Figure 1:**
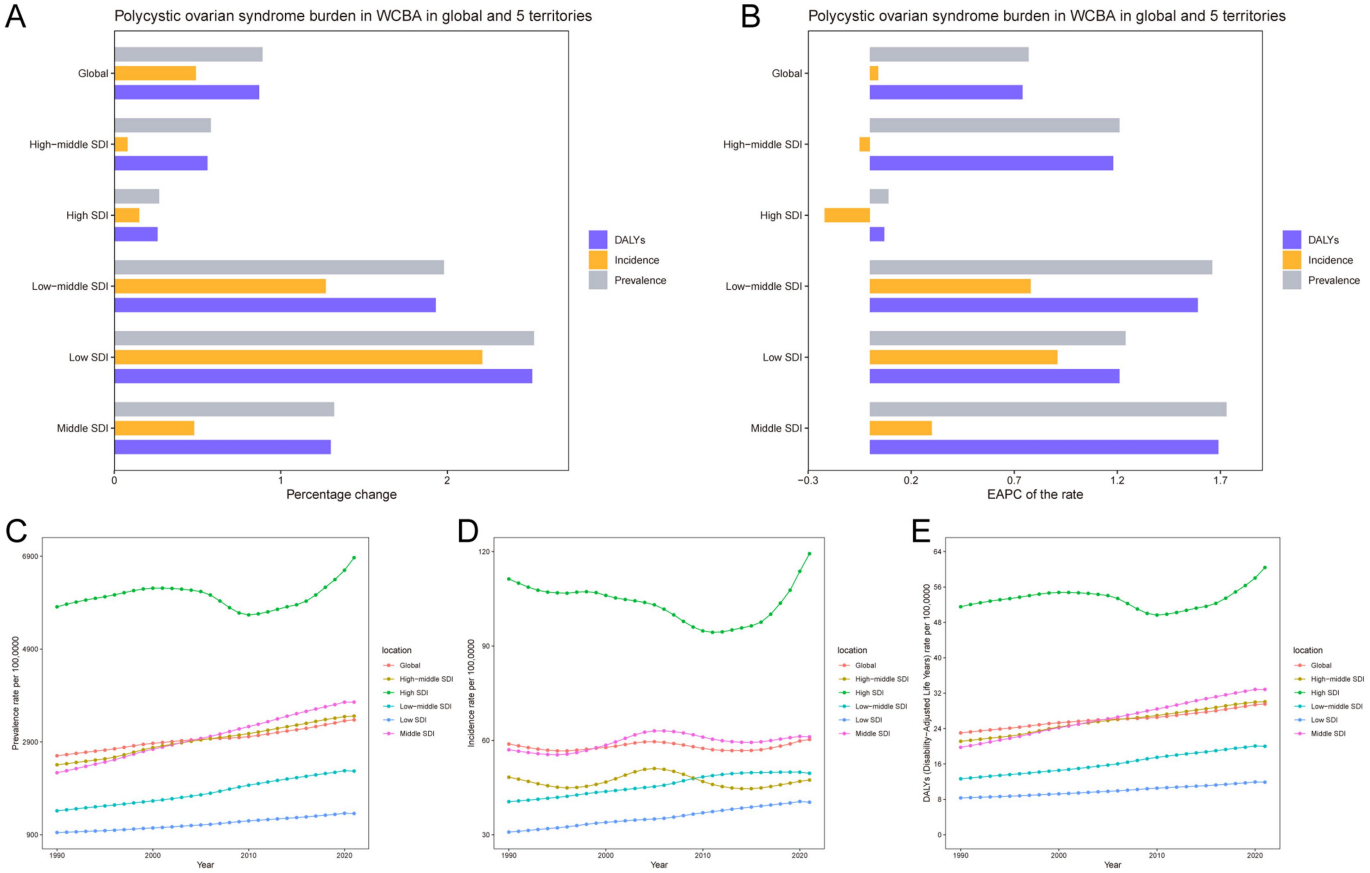
Temporal trend of polycystic ovary syndrome burden in WCBA in global and 5 territories. **(A)** Percentage change in cases of prevalent, incident, and DALYs in 1990 and 2021. **(B)** The EAPC of prevalence, incidence, and DALYs rates from 1990 to 2021. The rates of prevalence **(C)**, incidence **(D)**, and DALYs **(E)** from 1990 to 2021.

Globally, there has been a significant escalation in the number of prevalence, incidence, and DALYs cases associated with PCOS among WCBA (Table 1). The absolute number of prevalence cases of PCOS increased dramatically, with the number of prevalent cases soaring from 34.81 million in 1990 to 65.77 million in 2021, marking an 89% increase (Figure 1A). Similarly, the number of incident cases rose from 786.95 thousand in 1990 to 1,175.07 thousand in 2021, reflecting a 49% increase. The burden of the disease, as measured by DALYs, also saw a substantial rise, increasing from 307.94 thousand to 576.05 thousand, which corresponds to an 87% increase. Concurrently, the prevalence rates of PCOS among WCBA exhibited an increasing trend globally, with an EAPC of 0.77 (0.74–0.81; Figure 1B). Both incidence and DALYs also demonstrated an upward trend, with EAPCs of 0.04 (−0.03 to 0.11) and 0.74 (0.71 to 0.77), respectively. These findings indicate a growing number and burden of patients with PCOS worldwide (Supplementary Tables S1, S2).

**Table 1:** The prevalence of polycystic ovary syndrome cases and rates among WCBA in 1990 and 2021, and the trends from 1990 to 2021.

Location	1990_millions (95% UI)	2021_millions (95% UI)	Percentage change	1990_per 100,000 (95% UI)	2021_per 100,000 (95% UI)	EAPC (95% CI)
Andean Latin America	0.43 (0.3–0.6)	1.11 (0.76–1.55)	1.58	4568.87 (3146.35–6358.4)	6332.51 (4336.27–8867.7)	1.1 (1.03–1.17)
Australasia	0.43 (0.31–0.56)	0.67 (0.47–0.93)	0.56	7918.64 (5832.17–10416.61)	9213.66 (6561.16–12814.35)	0.27 (0.19–0.36)
Caribbean	0.21 (0.14–0.3)	0.34 (0.23–0.49)	0.62	2257.8 (1519.07–3247.57)	2825.76 (1918.97–4075.49)	0.75 (0.7–0.81)
Central Asia	0.11 (0.08–0.16)	0.23 (0.16–0.32)	1.09	667.59 (448.74–973.15)	929.26 (639.64–1304.2)	1.18 (1.1–1.27)
Central Europe	0.11 (0.07–0.16)	0.11 (0.08–0.16)	0	355.03 (235.42–530.72)	436.25 (297.91–615.3)	0.65 (0.6–0.7)
Central Latin America	2.13 (1.46–2.96)	3.81 (2.65–5.32)	0.79	5077.66 (3485.1–7054.77)	5582.44 (3878.65–7803.84)	−0.11 (−0.27–0.06)
Central Sub-Saharan Africa	0.1 (0.07–0.15)	0.42 (0.29–0.6)	3.2	843.96 (583.9–1228.21)	1281.7 (886.26–1849.84)	1.3 (1.15–1.44)
East Asia	5.39 (3.77–7.59)	9.87 (6.96–14.02)	0.83	1615.56 (1131.48–2276.32)	2983.92 (2103.55–4236.17)	2.06 (1.91–2.2)
Eastern Europe	0.22 (0.15–0.32)	0.25 (0.18–0.36)	0.14	401.47 (272.37–580.73)	520.31 (363.73–752.9)	1.04 (0.96–1.11)
Eastern Sub-Saharan Africa	0.42 (0.3–0.61)	1.36 (0.96–1.95)	2.24	979.72 (684.57–1412.07)	1272.44 (894.04–1817.39)	0.87 (0.84–0.91)
Global	34.81 (24.93–47.92)	65.77 (46.67–90.62)	0.89	2602.62 (1864.27–3583.12)	3374.68 (2394.97–4649.68)	0.77 (0.74–0.81)
High-income Asia Pacific	4.2 (3.03–5.85)	3.89 (2.75–5.44)	−0.07	9185.53 (6624.98–12792.03)	10239.02 (7234.28–14296.03)	0.29 (0.24–0.33)
High-income North America	4.29 (3.02–6)	6.07 (4.53–7.99)	0.41	5765.46 (4067.27–8071.03)	7225.93 (5394.79–9514.86)	−0.53 (−1.02--0.04)
High-middle SDI	6.69 (4.72–9.25)	10.55 (7.44–14.74)	0.58	2407.44 (1699.54–3331.25)	3456.66 (2437.81–4832.38)	1.21 (1.17–1.25)
High SDI	13.17 (9.56–18.42)	16.7 (12.28–22.71)	0.27	5810.11 (4218.38–8126.4)	6868.85 (5049.2–9340.23)	0.09 (−0.07–0.26)
Low-middle SDI	3.86 (2.71–5.42)	11.5 (8.01–16.19)	1.98	1416.12 (994.29–1985.78)	2272.04 (1581.38–3197.84)	1.66 (1.61–1.7)
Low SDI	1.06 (0.74–1.51)	3.73 (2.6–5.29)	2.52	948.52 (662.87–1356.22)	1358.55 (948.62–1927.76)	1.24 (1.21–1.27)
Middle SDI	10 (7.03–13.88)	23.24 (16.47–32.3)	1.32	2236.89 (1571.66–3104.37)	3758.17 (2662.53–5222.38)	1.73 (1.68–1.77)
North Africa and Middle East	2.31 (1.61–3.29)	6.34 (4.45–8.95)	1.74	2963.07 (2055.04–4208.84)	3975.8 (2791.13–5616.82)	1.11 (1.03–1.18)
Oceania	0.04 (0.03–0.05)	0.12 (0.08–0.17)	2	2443.52 (1681.99–3447.43)	3393.43 (2360.22–4846.83)	0.82 (0.66–0.99)
South Asia	3.11 (2.22–4.33)	10.75 (7.6–15.05)	2.46	1220.9 (869.54–1698.95)	2175.38 (1537.88–3046.4)	2.16 (2.04–2.29)
Southeast Asia	3.51 (2.48–4.94)	10 (7.06–14.1)	1.85	2917.33 (2061.92–4111.88)	5457.54 (3851.15–7695.68)	2.31 (2.21–2.41)
Southern Latin America	0.28 (0.2–0.41)	0.64 (0.45–0.92)	1.29	2276.47 (1573.89–3306.58)	3658.16 (2573.53–5251.35)	1.49 (1.28–1.7)
Southern Sub-Saharan Africa	0.22 (0.15–0.32)	0.46 (0.31–0.64)	1.09	1,657 (1142.33–2384.85)	2103.88 (1439.47–2969.21)	0.83 (0.76–0.91)
Tropical Latin America	0.42 (0.28–0.6)	0.69 (0.47–0.98)	0.64	1049.34 (710.46–1515.19)	1145.47 (777.94–1624.71)	−0.16 (−0.32–0.01)
Western Europe	6.46 (4.55–8.97)	7.01 (4.94–9.78)	0.09	6758.26 (4758.24–9390.76)	7518.71 (5296.66–10491.12)	0.2 (0.14–0.27)
Western Sub-Saharan Africa	0.41 (0.29–0.6)	1.64 (1.15–2.35)	3	951.13 (664.3–1,373)	1371.94 (959.89–1957.59)	0.93 (0.75–1.11)

### SDI regional level

In 2021, the highest number of prevalent, incident, and DALYs cases for PCOS among WCBA were observed in the middle SDI region, with 23.24 million (16.47–32.30), 378.02 thousand (251.37–556.46), and 203.16 thousand (90.25–427.04) cases, respectively. However, the highest prevalence, incidence, and DALYs rates were noted in the high SDI region (Figures 1C–E). Most SDI regions experienced an increasing trend in prevalence and DALYs rates from 1990 to 2021. The middle SDI region witnessed the most significant increase in prevalence, with an EAPC of 1.73 (1.68–1.77). In contrast, the high SDI and high-middle SDI regions showed a decrease in incidence, with EAPCs of −0.22 (−0.43 to −0.01) and − 0.05 (−0.21 to −0.10), respectively. The middle SDI region also experienced the most pronounced rise in DALYs, with an EAPC of 1.69 (1.64–1.74).

### GBD regional level

**Figure 2:**
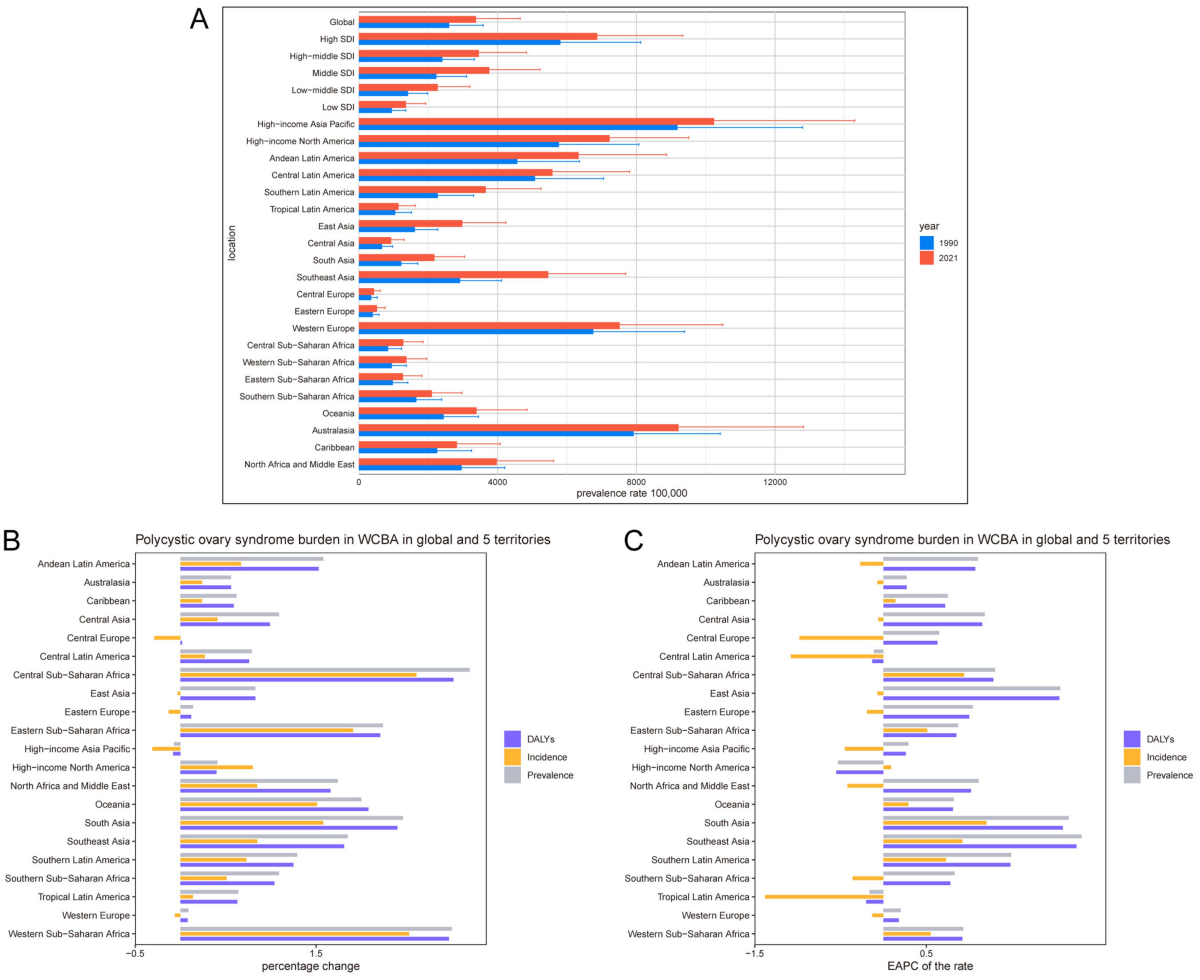
Temporal trend of polycystic ovary syndrome burden in WCBA in regions. **(A)** Prevalence rate per 100,000 population in 1990 and 2021. **(B)** Percentage change in cases of prevalent, incident, and DALYs in 1990 and 2021. **(C)** EAPC of rates of prevalent, incident, and DALYs from 1990 to 2021.

Over the past 32 years, the number of prevalent and incident cases of PCOS among WCBA increased in most of the 21 GBD regions, with the most notable increase occurring in Central Sub-Saharan Africa, where percentage changes were 320 and 260%, respectively (Figures 2A,B; Supplementary Figures S1, S2). High-income Asia Pacific was an exception, showing a decline in prevalence and incidence cases, with percentage changes of −7% and − 31%, respectively. Similarly, the number of DALY cases decreased only in the High-income Asia Pacific region, with a percentage change of −8%, while all other regions experienced an increase. At the same time, only three GBD regions showed a decrease in prevalence rates, with High-income North America showing the highest decrease, with an EAPC of −0.53 (−1.02 to −0.04), and Southeast Asia showing the highest increase, with an EAPC of 2.31 (2.21–2.41). Seven regions showed an increase in incidence rates, with South Asia exhibiting the highest increase, with an EAPC of 1.20 (1.10–1.30). Fourteen regions experienced a decrease in incidence rates, with Tropical Latin America experiencing the highest decrease, with an EAPC of −1.38 (−1.60 to −1.17). Three regions experienced declines in DALY rates, with High-income North America experiencing the most significant decline, with an EAPC of −0.55 (−1.03 to −0.06). Southeast Asia experienced the most significant increase, with an EAPC of 2.25 (2.16–2.35; Figure 2C).

### Country level

**Figure 3:**
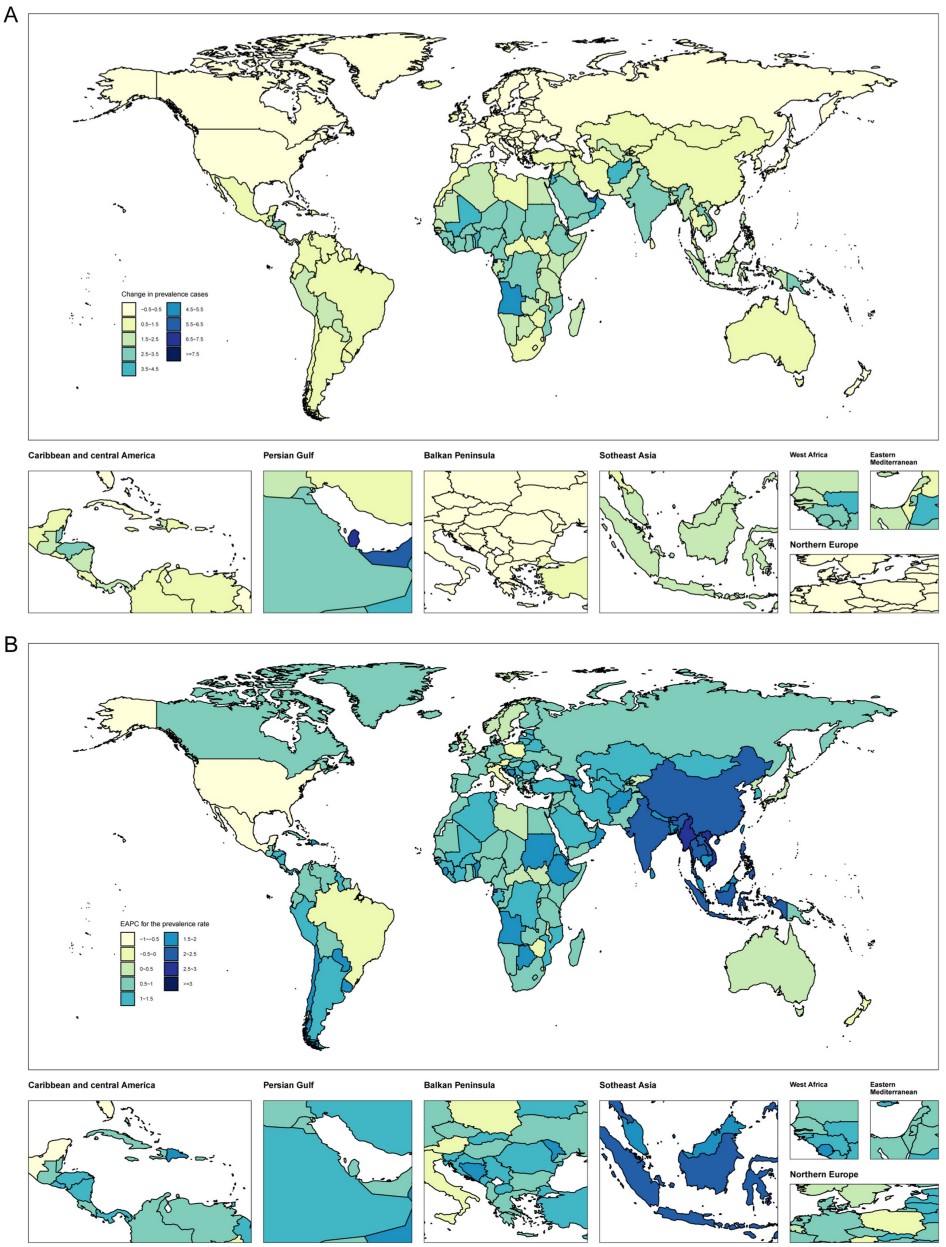
Temporal trend of polycystic ovary syndrome burden in WCBA globally. **(A)** Percentage change in prevalent cases across 204 countries in 1990 and 2021. **(B)** EAPC in prevalent rates across 204 countries from 1990 to 2021.

From 1990 to 2021, the number of prevalent, incident, and DALY cases among WCBA increased in most countries. Equatorial Guinea experienced the highest increase in the number of prevalent and incident cases, with percentage changes of 776 and 571%, respectively (Figure 3A; Supplementary Figure S3). The United States Virgin Islands presented the highest decrease in prevalence cases, with a percentage change of −25%. Italy recorded the greatest decrease in incidence cases, with a percentage change of −41%. Equatorial Guinea also had the greatest increase in DALYs, with a percentage change of 777%, while the United States Virgin Islands had the highest decrease, with a percentage change of −25%. Most countries exhibited an increasing trend in prevalence, incidence, and DALY rates (Figure 3B; Supplementary Figures S4–S6; Supplementary Tables S3–S5).

### Age patterns

**Figure 4:**
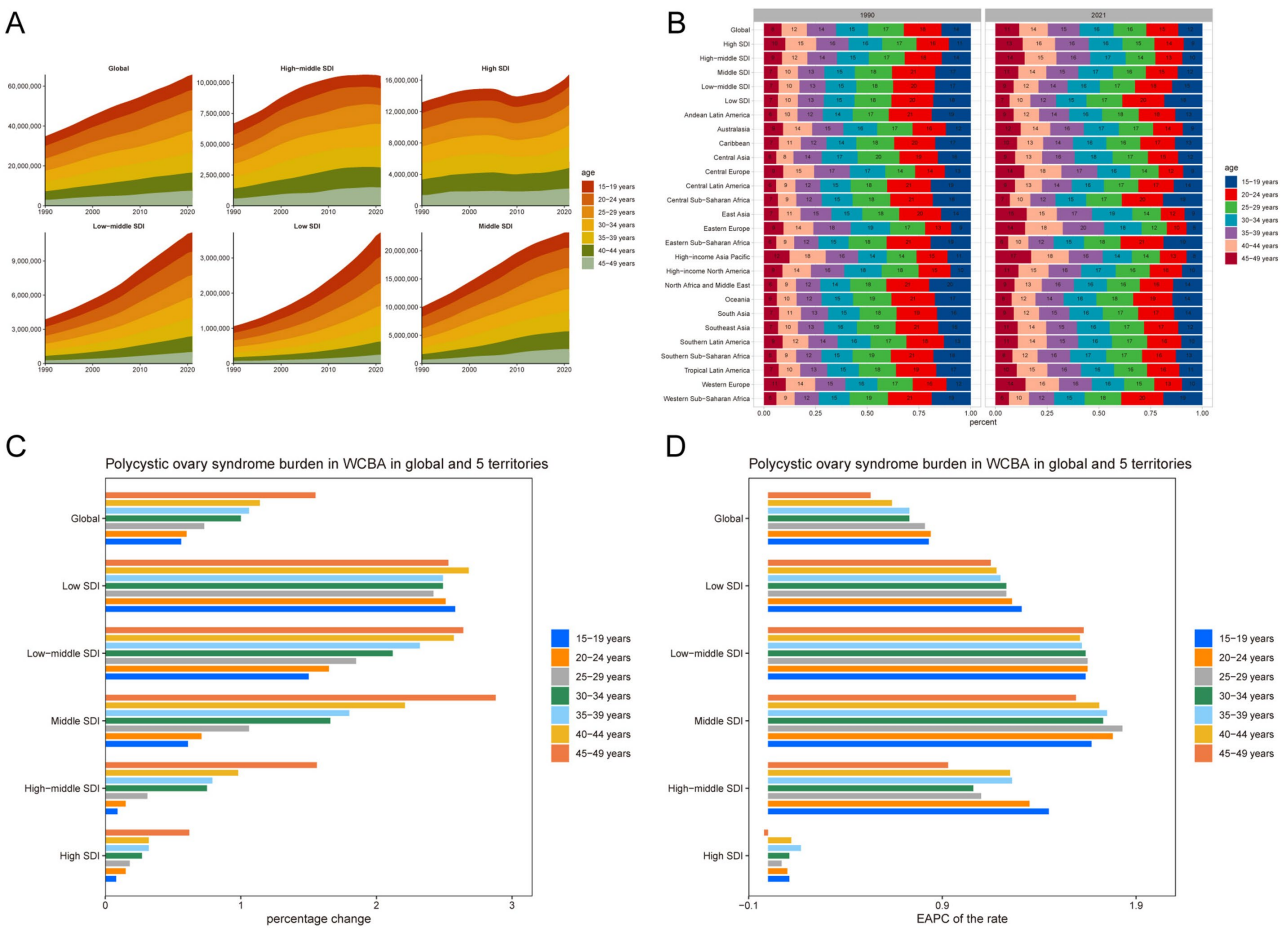
Temporal trend of polycystic ovary syndrome burden in WCBA by age pattern in different regions. **(A)** Prevalent cases of 7 age groups (15–49 years, 5-year intervals) from 1990 to 2021 globally and in 5 territories (low to high SDI). **(B)** The distribution of prevalent cases across 7 age groups as percentages globally, in 5 territories, and 21 GBD regions in 1990 and 2021. **(C)** Percentage change in prevalent cases of 7 age groups globally and in 5 territories in 1990 and 2021. **(D)** EAPC of prevalent rates of 7 age groups globally and in 5 territories from 1990 to 2021.

From 1990 to 2021, there was a notable increase in the prevalence, incidence and DALYs cases of PCOS among WCBA worldwide, with the most significant changes observed in the 45–49 age group (Figures 4A,B; Supplementary Figures S7, S8; Table 2; Supplementary Tables S6, S7). The prevalence cases presented a 154% increase (1.54–1.53), while the incidence and DALYs cases increased by 114% (1.09–1.13) and 154% (1.54–1.53) respectively, as depicted in Figure 4C and Supplementary Figures S9, S10. Moreover, on a global scale, the incidence rates of PCOS among WCBA have exhibited a marked upward trend, particularly in the 15–19 age group, with an EAPC of 0.73 (0.69–0.77; Supplementary Figure S11).

**Table 2:** The prevalence of polycystic ovary syndrome cases and rates among WCBA in 1990 and 2021, and the trends in age patterns from 1990 to 2021.

Location	Age	1990_millions (95% UI)	2021_millions (95% UI)	Percentage change	1990_per 100,000 (95% UI)	2021_per 100,000 (95% UI)	EAPC (95% CI)
Global	15–19 years	4.92 (3.34–7.02)	7.66 (5.18–10.97)	0.56 (0.55–0.56)	1925.13 (1305.23–2746.17)	2523.76 (1707–3613.35)	0.83 (0.81–0.86)
Global	15–49 years	34.81 (24.93–47.92)	65.77 (46.67–90.62)	0.89 (0.87–0.89)	2602.62 (1864.27–3583.12)	3374.68 (2394.97–4649.68)	0.77 (0.74–0.81)
Global	20–24 years	6.35 (4.51–8.84)	10.15 (7.31–13.93)	0.6 (0.62–0.58)	2600.45 (1849.19–3620.8)	3453.97 (2489.13–4742.61)	0.84 (0.8–0.88)
Global	25–29 years	6.03 (4.32–8.44)	10.42 (7.48–14.46)	0.73 (0.73–0.71)	2740.3 (1962.1–3836.74)	3581.86 (2571.9–4968.04)	0.81 (0.76–0.86)
Global	30–34 years	5.37 (3.85–7.48)	10.76 (7.71–14.97)	1 (1–1)	2824.88 (2025.68–3934.06)	3600.5 (2579.79–5007.28)	0.73 (0.7–0.76)
Global	35–39 years	4.93 (3.52–6.84)	10.13 (7.19–14.08)	1.05 (1.04–1.06)	2840.34 (2026.55–3941.19)	3644.97 (2588.05–5067.78)	0.73 (0.69–0.78)
Global	40–44 years	4.26 (3.08–5.94)	9.15 (6.54–12.71)	1.15 (1.12–1.14)	3041.51 (2196.24–4233.57)	3687.03 (2637.99–5121.84)	0.64 (0.58–0.7)
Global	45–49 years	2.95 (2.12–4.07)	7.5 (5.41–10.38)	1.54 (1.55–1.55)	2587.93 (1859.21–3573.47)	3182.5 (2297.85–4407.06)	0.53 (0.46–0.59)
Low SDI	15–19 years	0.19 (0.12–0.29)	0.69 (0.45–1.01)	2.63 (2.75–2.48)	764.63 (488.84–1154.33)	1114.56 (736.65–1638.8)	1.31 (1.28–1.34)
Low SDI	15–49 years	1.06 (0.74–1.51)	3.73 (2.6–5.29)	2.52 (2.51–2.5)	948.52 (662.87–1356.22)	1358.55 (948.62–1927.76)	1.24 (1.21–1.27)
Low SDI	20–24 years	0.21 (0.14–0.31)	0.75 (0.52–1.09)	2.57 (2.71–2.52)	987.26 (667.36–1429.66)	1429.3 (978.4–2059.66)	1.26 (1.24–1.29)
Low SDI	25–29 years	0.19 (0.13–0.27)	0.65 (0.45–0.92)	2.42 (2.46–2.41)	1020.92 (705.26–1453.39)	1467.6 (1023.7–2078.47)	1.23 (1.21–1.25)
Low SDI	30–34 years	0.16 (0.11–0.22)	0.55 (0.38–0.77)	2.44 (2.45–2.5)	1029.61 (721.48–1451.07)	1472.48 (1023.11–2075.44)	1.23 (1.21–1.25)
Low SDI	35–39 years	0.13 (0.09–0.19)	0.47 (0.33–0.65)	2.62 (2.67–2.42)	1037.02 (733.51–1451.94)	1461.59 (1020.2–2043.49)	1.2 (1.17–1.24)
Low SDI	40–44 years	0.1 (0.07–0.14)	0.38 (0.27–0.53)	2.8 (2.86–2.79)	1030.85 (731.75–1432.36)	1438.53 (1017.28–2011.31)	1.18 (1.14–1.22)
Low SDI	45–49 years	0.07 (0.05–0.1)	0.25 (0.18–0.35)	2.57 (2.6–2.5)	857.99 (614.82–1180.79)	1209.61 (853.76–1679.29)	1.15 (1.11–1.2)
Low-middle SDI	15–19 years	0.67 (0.44–0.98)	1.68 (1.13–2.46)	1.51 (1.57–1.51)	1148.2 (755.38–1664.84)	1861.98 (1251.2–2717.47)	1.64 (1.6–1.68)
Low-middle SDI	15–49 years	3.86 (2.71–5.42)	11.5 (8.01–16.19)	1.98 (1.96–1.99)	1416.12 (994.29–1985.78)	2272.04 (1581.38–3197.84)	1.66 (1.61–1.7)
Low-middle SDI	20–24 years	0.78 (0.53–1.11)	2.06 (1.43–2.91)	1.64 (1.7–1.62)	1489.83 (1017.48–2126.8)	2369.84 (1646.63–3340.64)	1.65 (1.6–1.7)
Low-middle SDI	25–29 years	0.69 (0.48–0.97)	1.96 (1.38–2.75)	1.84 (1.87–1.84)	1530.26 (1068.37–2153.04)	2411.36 (1700.95–3381.26)	1.65 (1.59–1.7)
Low-middle SDI	30–34 years	0.58 (0.41–0.81)	1.79 (1.27–2.53)	2.09 (2.1–2.12)	1536.68 (1090.06–2153.51)	2427.56 (1718.88–3422.65)	1.64 (1.58–1.7)
Low-middle SDI	35–39 years	0.49 (0.35–0.68)	1.62 (1.14–2.29)	2.31 (2.26–2.37)	1520.66 (1079.29–2120.64)	2427.97 (1713.35–3439.5)	1.62 (1.58–1.67)
Low-middle SDI	40–44 years	0.39 (0.28–0.54)	1.38 (0.97–1.95)	2.54 (2.46–2.61)	1496.59 (1065.89–2087.18)	2399.8 (1687.65–3379.6)	1.61 (1.58–1.63)
Low-middle SDI	45–49 years	0.28 (0.2–0.38)	1 (0.72–1.42)	2.57 (2.6–2.74)	1269.68 (912.44–1760.9)	2029.23 (1463.55–2880.38)	1.63 (1.6–1.66)
Middle SDI	15–19 years	1.72 (1.15–2.48)	2.77 (1.86–3.96)	0.61 (0.62–0.6)	1863.87 (1249.84–2686.41)	3151.36 (2115.99–4513.3)	1.67 (1.61–1.72)
Middle SDI	15–49 years	10 (7.03–13.88)	23.24 (16.47–32.3)	1.32 (1.34–1.33)	2236.89 (1571.66–3104.37)	3758.17 (2662.53–5222.38)	1.73 (1.68–1.77)
Middle SDI	20–24 years	2.09 (1.48–2.91)	3.57 (2.52–4.89)	0.71 (0.7–0.68)	2367.73 (1676.6–3296.87)	4123.9 (2910.31–5645.79)	1.78 (1.7–1.86)
Middle SDI	25–29 years	1.8 (1.28–2.49)	3.71 (2.62–5.13)	1.06 (1.05–1.06)	2404.76 (1704.06–3319.11)	4095.72 (2890.34–5659.53)	1.83 (1.74–1.91)
Middle SDI	30–34 years	1.47 (1.04–2.04)	3.9 (2.75–5.43)	1.65 (1.64–1.66)	2441.65 (1732.51–3389.65)	3945.58 (2788.11–5503.28)	1.73 (1.66–1.81)
Middle SDI	35–39 years	1.28 (0.91–1.78)	3.59 (2.54–5.02)	1.8 (1.79–1.82)	2314.74 (1641.3–3212.55)	3913.19 (2766.77–5479.65)	1.75 (1.7–1.8)
Middle SDI	40–44 years	0.98 (0.69–1.36)	3.14 (2.23–4.41)	2.2 (2.23–2.24)	2315.1 (1640.7–3223.36)	3840.77 (2723.62–5393.05)	1.71 (1.65–1.78)
Middle SDI	45–49 years	0.66 (0.47–0.92)	2.56 (1.82–3.59)	2.88 (2.87–2.9)	1952.08 (1396.2–2710.42)	3160.82 (2244.16–4425.16)	1.59 (1.54–1.65)
High-middle SDI	15–19 years	0.94 (0.63–1.35)	1.02 (0.68–1.46)	0.09 (0.08–0.08)	1976 (1326.53–2843.95)	2956.18 (1975.09–4236.57)	1.45 (1.34–1.56)
High-middle SDI	15–49 years	6.69 (4.72–9.25)	10.55 (7.44–14.74)	0.58 (0.58–0.59)	2407.44 (1699.54–3331.25)	3456.66 (2437.81–4832.38)	1.21 (1.17–1.25)
High-middle SDI	20–24 years	1.2 (0.85–1.66)	1.38 (0.97–1.91)	0.15 (0.14–0.15)	2491.82 (1764.69–3446.52)	3872.06 (2735.79–5372.94)	1.35 (1.26–1.45)
High-middle SDI	25–29 years	1.16 (0.83–1.6)	1.52 (1.07–2.12)	0.31 (0.29–0.32)	2538.13 (1820.78–3497.72)	3767.15 (2657.62–5258.55)	1.1 (0.98–1.21)
High-middle SDI	30–34 years	1.03 (0.74–1.42)	1.8 (1.27–2.53)	0.75 (0.72–0.78)	2468.93 (1768.11–3390.55)	3498.34 (2461.08–4906.44)	1.06 (0.91–1.21)
High-middle SDI	35–39 years	0.96 (0.69–1.32)	1.72 (1.22–2.43)	0.79 (0.77–0.84)	2431.83 (1739.17–3347.92)	3476.76 (2466.22–4905.98)	1.26 (1.11–1.41)
High-middle SDI	40–44 years	0.82 (0.59–1.14)	1.62 (1.15–2.27)	0.98 (0.95–0.99)	2664.49 (1917.56–3702.97)	3565.05 (2524.38–4994.65)	1.25 (1.15–1.34)
High-middle SDI	45–49 years	0.58 (0.41–0.81)	1.49 (1.06–2.08)	1.57 (1.59–1.57)	2,365 (1687.45–3289.75)	3080.46 (2196.64–4308.81)	0.93 (0.82–1.05)
High SDI	15–19 years	1.39 (0.97–1.99)	1.5 (1.06–2.1)	0.08 (0.09–0.06)	4376.36 (3037.26–6242.37)	5160.06 (3644.03–7206.35)	0.11 (−0.06–0.27)
High SDI	15–49 years	13.17 (9.56–18.42)	16.7 (12.28–22.71)	0.27 (0.28–0.23)	5810.11 (4218.38–8126.4)	6868.85 (5049.2–9340.23)	0.09 (−0.07–0.26)
High SDI	20–24 years	2.06 (1.49–2.83)	2.37 (1.76–3.2)	0.15 (0.18–0.13)	6139.9 (4444.26–8417.83)	7519.96 (5583.76–10152.72)	0.1 (−0.09–0.29)
High SDI	25–29 years	2.19 (1.61–3.06)	2.58 (1.9–3.52)	0.18 (0.18–0.15)	6106.27 (4484.85–8535.4)	7466.98 (5489.29–10184.44)	0.07 (−0.14–0.27)
High SDI	30–34 years	2.14 (1.55–2.96)	2.72 (2.02–3.66)	0.27 (0.3–0.24)	6025.42 (4376.03–8353.83)	7259.07 (5399.02–9781.51)	0.11 (−0.07–0.29)
High SDI	35–39 years	2.06 (1.47–2.87)	2.73 (2.01–3.77)	0.33 (0.37–0.31)	6158.14 (4385.59–8575.04)	7190.32 (5292.13–9928.82)	0.17 (0.04–0.29)
High SDI	40–44 years	1.97 (1.43–2.77)	2.61 (1.93–3.62)	0.32 (0.35–0.31)	6323.47 (4583.14–8883.54)	7121.68 (5257.84–9866.55)	0.12 (−0.02–0.25)
High SDI	45–49 years	1.35 (0.98–1.89)	2.19 (1.61–2.98)	0.62 (0.64–0.58)	5362.68 (3862.38–7488.44)	6101.08 (4489.05–8300.11)	−0.02 (−0.22–0.18)

Over the past three decades, the incidence cases of PCOS among WCBA in low SDI regions has experienced the most substantial increase. However, different age groups display distinct patterns. The middle SDI region, particularly the 45–49 age group, reported the highest percentage increase in prevalence cases, approximately 288%. The low SDI region, with the 15–19 age group, presented the highest percentage increase in incidence cases, around 241%. Similarly, the middle SDI region, with the 45–49 age group, had the highest percentage increase in DALYs cases, about 287%.

Significantly, across the five SDI regions, the rates of PCOS prevalence, incidence, and DALYs among WCBA have shown a significant upward trend. However, in the high SDI region, a decreasing trend was observed for prevalence, incidence, and DALYs rates in the 45–49 age group, with an EAPC of −0.02 (−0.22 to 0.18), −0.32 (−0.47 to −0.17), and − 0.02 (−0.22 to 0.17) respectively (Figure 4D; Supplementary Figures S11, S12). The middle SDI region, with the 25–29 age group, had the highest prevalence and DALYs rates, with an EAPC of 1.83 (1.74–1.91) and 1.81 (1.72–1.90) respectively. The middle SDI region, with the 15–19 age group, had the highest incidence rates, with an EAPC of 1.58 (1.53–1.64).

Globally, over the past 32 years, the 45–49 age group has seen the fastest growth in prevalence cases of PCOS, representing about 11% of all age groups, with a 3% increase. Among the five SDI regions, the high-middle SDI region has experienced the fastest growth in PCOS cases in the 45–49 age group, accounting for approximately 14% of all age groups, with a 5% increase. In the 21 GBD SDI regions, the High-income Asia Pacific region has shown the fastest growth in PCOS cases in the 45–49 age group, representing about 17% of all age groups, with a 5% increase. Moreover, the 15–19 age group is identified as the peak age for the onset of PCOS, followed by the 20–24 age group (Supplementary Figures S13, S14).

### Association between PCOS burden and SDI

**Figure 5:**
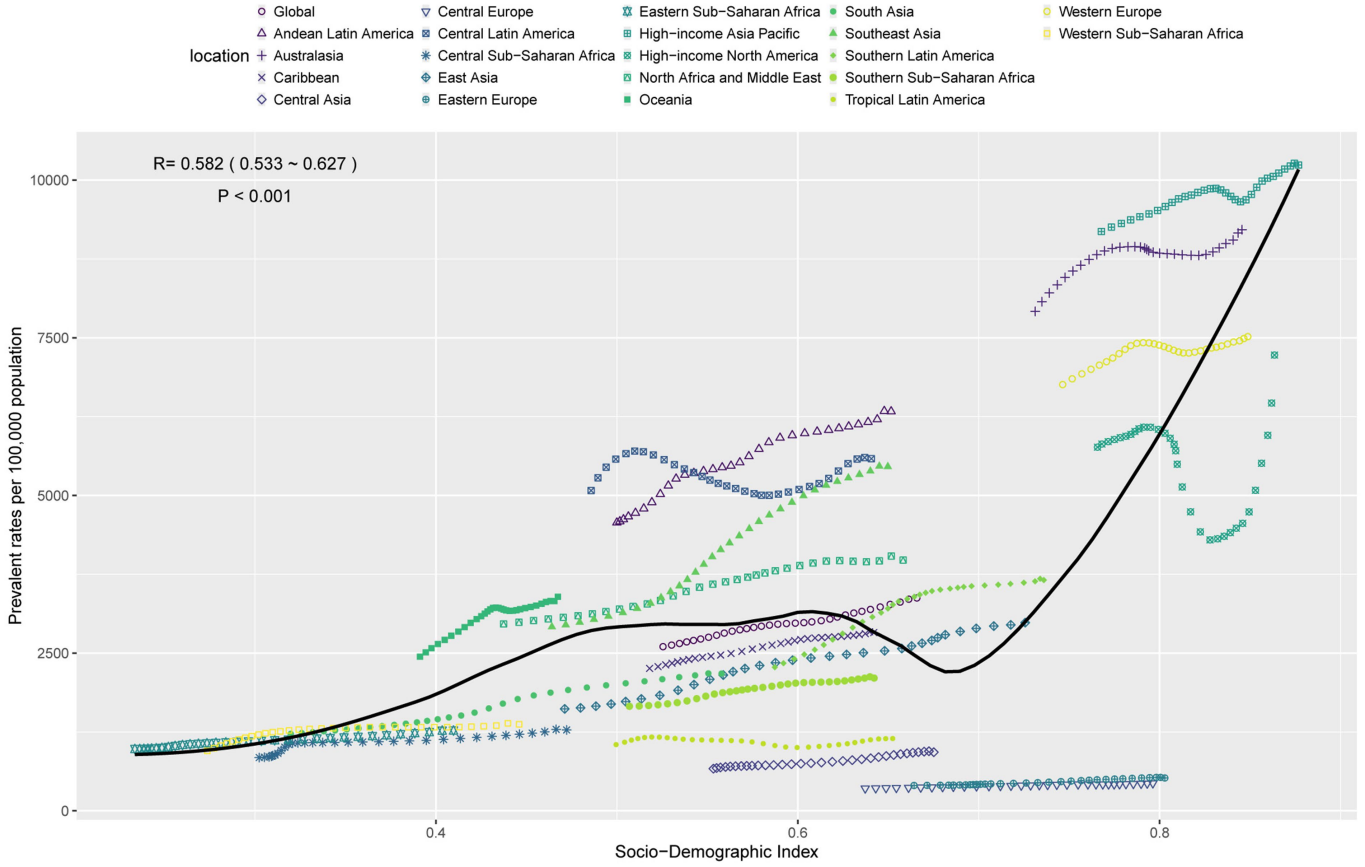
The associations between the SDI and prevalent rates per 100,000 population of polycystic ovary syndrome in WCBA across 21 GBD regions.

There was a significant relationship between the prevalence of PCOS and SDI in 2021, with a correlation coefficient of *r* = 0.582 and *p* < 0.001, indicating that this correlation was statistically significant (Figure 5). Overall, the prevalence rates of PCOS increased with the increase in SDI, suggesting that the prevalence rates of PCOS is higher in areas with higher levels of economic and social development. This trend may reflect greater access to diagnosis, higher probability of exposure to certain risk factors (e.g., obesity or cardiovascular disease), or lifestyle-related factors in high-income countries. The burden of PCOS is relatively stable in areas with SDI values between 0.5 and 0.6. However, the burden of disease increases significantly when the SDI reaches 0.7 and above. High-income countries or regions such as Australasia and high-income Asia Pacific have a higher burden of PCOS than predicted by the model, suggesting that these regions may have higher risk factors or higher diagnosis rates. Conversely, some low- and middle-income regions such as Central Asia and Central Europe have a lower-than-predicted burden of PCOS, which may be due to lifestyle, genetic factors, or other unknown protective factors in these regions (Supplementary Figures S15, S16).

## Discussion

PCOS is a prevalent endocrine disorder affecting WCBA, with global prevalence estimates ranging from 5 to 10% (28). The onset of PCOS is typically during the peripubertal period, and its complex etiology involves genetic, developmental, neuroendocrine, inflammatory, microbiome, and environmental factors (29–31). While the exact pathogenesis remains elusive, it is linked to hypothalamic–pituitary-ovarian axis dysfunction, androgen excess, and insulin resistance (32). In China, PCOS affects approximately 5.6% of reproductive-age women, contributing significantly to anovulatory infertility (33). Common symptoms include menstrual irregularities, hirsutism, infertility, and hyperandrogenism. Additionally, PCOS is associated with increased risks of insulin resistance, type 2 diabetes, dyslipidemia, cardiovascular diseases, metabolic syndrome, sleep apnea, non-alcoholic fatty liver disease, gestational diabetes, and pregnancy-related hypertensive disorders (3, 23). The prevalence of this condition varies with ethnicity, geography, study populations, and diagnostic criteria. A 2017 global study reported 1.55 million incidence cases, 0.43 million DALYs, and an incidence rate of 82.44 per 100,000, marking a 1.45% increase since 2007 (3, 34). A comprehensive analysis of 32 years of GBD data reveals the burden of PCOS in global, regional, and national WCBA populations and explores its correlation with the SDI.

From 1990 to 2021, the WCBA population saw an 89% increase in PCOS prevalence, a 49% rise in incidence, and an 87% surge in DALYs, indicating a substantial disease burden. Diagnosis rates have increased significantly, driven by enhanced medical resource allocation, greater awareness of female health issues, and widespread implementation of the Rotterdam diagnostic criteria. However, the obesity epidemic has also contributed to PCOS incidence, with obese women having two to three times the risk of non-obese women. Genetic factors significantly influence PCOS development, often presenting a familial pattern.

Insulin resistance and obesity are identified as pivotal in PCOS pathogenesis, affecting approximately 60% and 50–75% of patients, respectively (35). Poor diet and sedentary lifestyles exacerbate these risks (36). SDI changes reflect socio-economic and lifestyle impacts on PCOS incidence, with middle SDI countries showing the highest increase in incidence rates and high-middle SDI countries in DALYs rates. Racial differences in incidence and diagnosis should not be overlooked.

In 2021, the disease burden of PCOS among WCBA globally exhibited some key trends. Across different SDI regions, countries with a middle SDI had the highest prevalence, incidence, and DALYs cases for PCOS. However, high SDI countries topped the charts in terms of prevalence, incidence, and DALY rates. From 1990 to 2021, there was an upward trend in prevalence and DALY rates in most SDI regions, with a particularly significant increase in prevalence in middle SDI countries. Despite this, the incidence in high SDI and high-middle SDI countries has declined. In terms of DALY rates, the most notable increase was also seen in middle SDI countries. These data reveal that while the global disease burden of PCOS has generally increased, there are significant differences in the growth trends and magnitude of change across different SDI countries. High SDI countries have the highest prevalence and DALY rates, while middle SDI countries have the fastest growth rate, which may reflect the impact of socio-economic development and lifestyle changes on the incidence of PCOS. Additionally, the decline in incidence rates in some high SDI countries may be related to the allocation of medical resources and disease management strategies in these countries.

Over 32 years, PCOS incidence and prevalence have generally increased, especially in Central Africa, with a 320 and 260% rise, respectively. The high-income Asia-Pacific region alone showed a downward trend, with a 7% decrease in prevalence and a 31% drop in incidence, and a reduction in DALYs. Regional variations in prevalence and incidence changes were observed, with Southeast Asia showing the most significant increase and Tropical Latin America the most decrease. DALY rate changes also varied, with high-income North America showing the most significant decrease and Southeast Asia the most increase.

From 1990 to 2021, at the national level, the prevalence, incidence, and DALY cases of PCOS have increased in most countries. During this period, Equatorial Guinea saw the most significant surge in the number of cases for both prevalence and incidence, with increases of 776 and 571%, respectively. Meanwhile, the US Virgin Islands experienced the most notable decrease in prevalence, while Italy led in the reduction of incidence. In terms of DALY increase, Equatorial Guinea topped the list, and the US Virgin Islands was at the forefront of reduction. Overall, the upward trend in these three key indicators in most countries highlights the significant challenge that PCOS poses to the health of women globally, particularly in Central Africa and the sub-Saharan region as well as Southeast Asia. Concurrently, the downward trend in the high-income Asia-Pacific region indicates the importance of continued efforts to maintain this positive trend. These trends reveal the impact of healthcare services and public health strategies on the management of PCOS in different regions, emphasizing the need for targeted medical resource allocation and public health strategies to address this challenge.

From 1990 to 2021, the prevalence and incidence rates of PCOS among WCBA globally increased significantly with age, especially in the 45 to 49 age group. Globally, the incidence rates of PCOS are on the rise, with the greatest increase seen in the 15 to 19-year-old age group. Changes in different SDI regions have their own characteristics. The increase in incidence rates is particularly pronounced in low-SDI areas, especially in younger age groups. In middle SDI areas, older women have a significant increase in disease burden. The only exception was the high SDI area, where disease burden showed a downward trend in the 45 to 49 age group. Overall, the 45 to 49-year-old age group is the group with the fastest growing PCOS cases. The growth rate is particularly obvious in the high-middle SDI region, and the growth rate is also particularly prominent in the high-income Asia-Pacific region. The peak incidence of PCOS is mainly between the ages of 15 and 19, followed by those between the ages of 20 and 24.

This study, while insightful, is not without its limitations. Firstly, the precision of the GBD data is contingent upon the caliber and accessibility of data from a multitude of nations, which can lead to inconsistencies in data gathering and reporting. The GBD dataset is predicated on health system information that may be incomplete or erroneous in certain regions, notably in low-income areas where healthcare infrastructure is lacking. Secondly, there is a variation in diagnostic criteria for PCOS across different regions, such as those established by the NIH, Rotterdam, and the Androgen Excess and PCOS Society. These diverse criteria can lead to heterogeneity in the rates of prevalence and incidence. It is recommended that future research strive to harmonize diagnostic standards across regions to facilitate more precise comparisons. Thirdly, although this study concentrates on the global trends and impact of PCOS, it does not delve deeply into the multifaceted etiology of the condition. PCOS is shaped by an interplay of genetic, environmental, and lifestyle factors, aspects that were not thoroughly examined in this analysis. Subsequent studies should endeavor to explore the interplay among these factors to gain a more holistic understanding of the development and progression of PCOS.

## Conclusion

Over the past 32 years, the global burden of PCOS among WCBA has increased significantly, peaking in the 45–49 age group, particularly in middle SDI regions. The positive correlation between prevalence and SDI highlights the crucial role of socio-economic factors in the epidemiology of PCOS. The physiological characteristics of WCBA make them more susceptible to PCOS, emphasizing the importance of tailored intervention strategies across different age groups and regions. Enhancing early diagnosis, prevention, and comprehensive management of PCOS, with a special focus on the physiological and psychological health of WCBA, is essential for reducing the global burden of PCOS and improving the quality of life for women.

## Data Availability

The original contributions presented in the study are included in the article/Supplementary material, further inquiries can be directed to the corresponding authors.
