# Supplementary material for: Global burden of polycystic ovary syndrome among women of childbearing age, 1990–2021: a systematic analysis using the global burden of disease study 2021
Source: Front Public Health. 2025 Mar 26;13:1514250. doi: 10.3389/fpubh.2025.1514250 (PMC11979288; doi:10.3389/fpubh.2025.1514250)
Supplement: Supplementary file 1 [file Data_Sheet_1.pdf]

Supplementary Figure

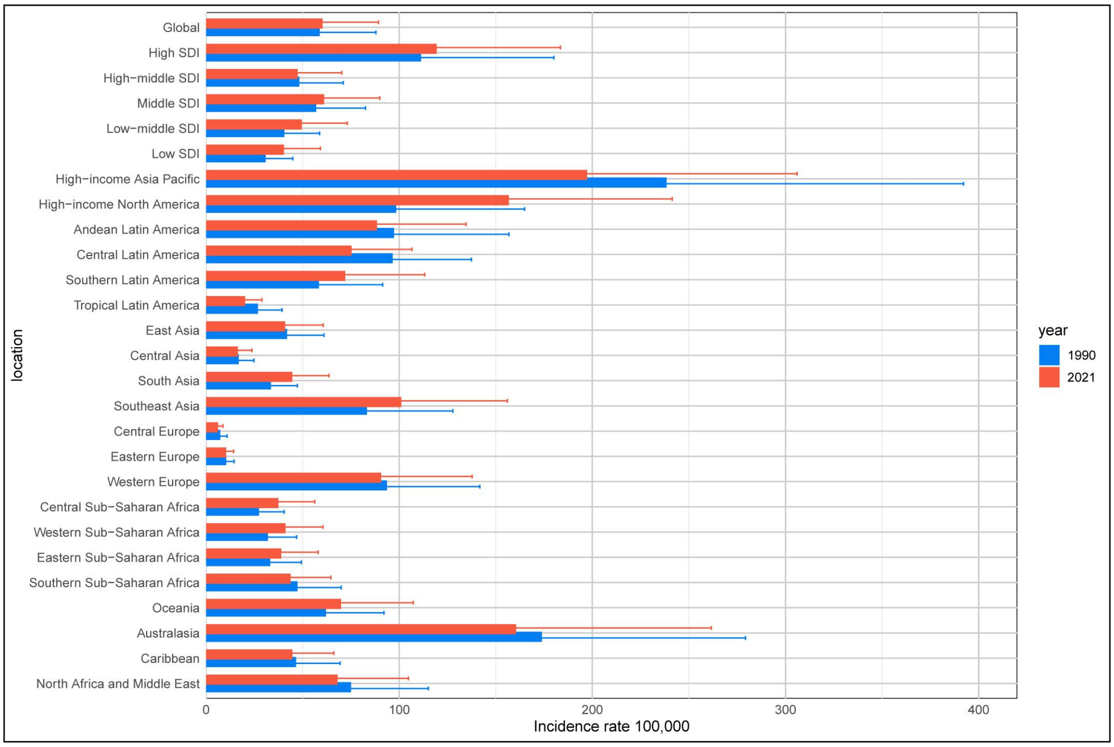

Supplementary Figure 1. Incidence rate per 100,000 population in 1990 and 2021.

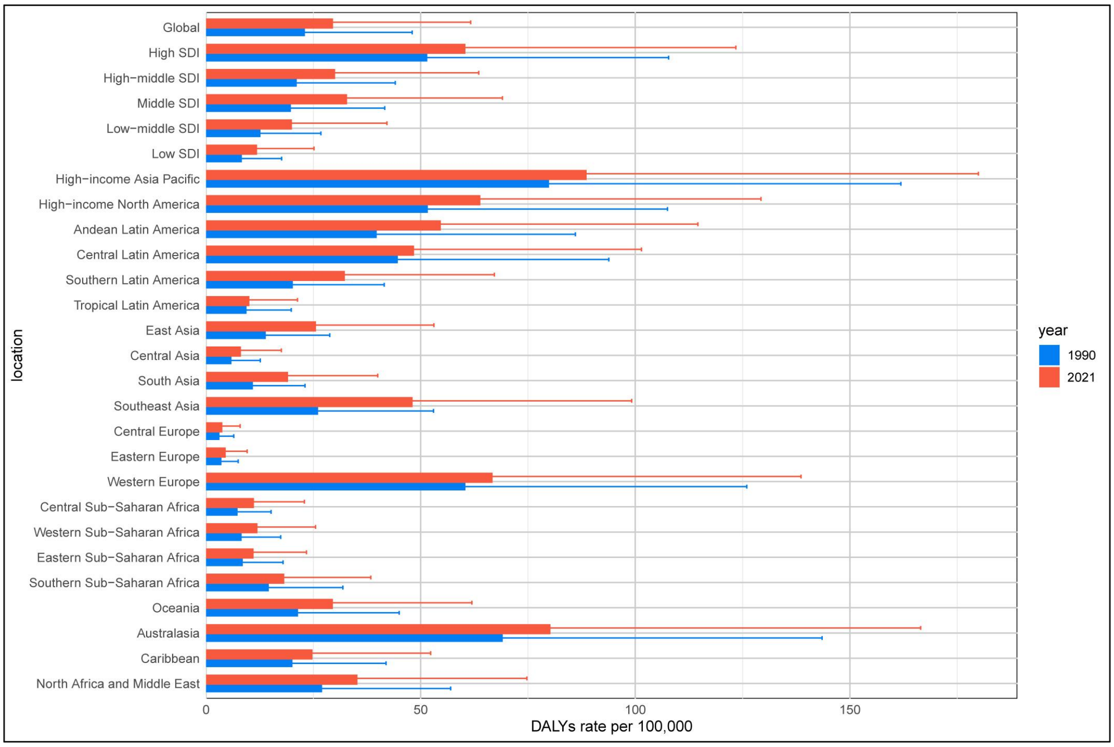

Supplementary Figure 2. DALYs rate per 100,000 population in 1990 and 2021.

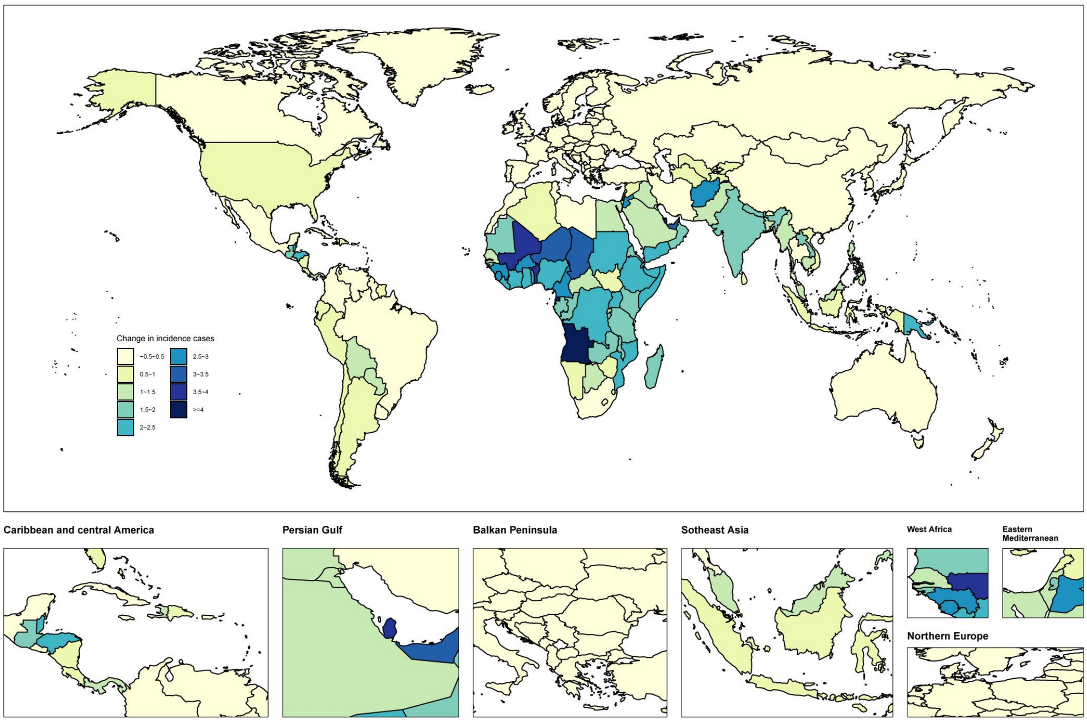

Supplementary Figure 3. Percentage change in incidence cases across 204 countries in 1990 and 2021.

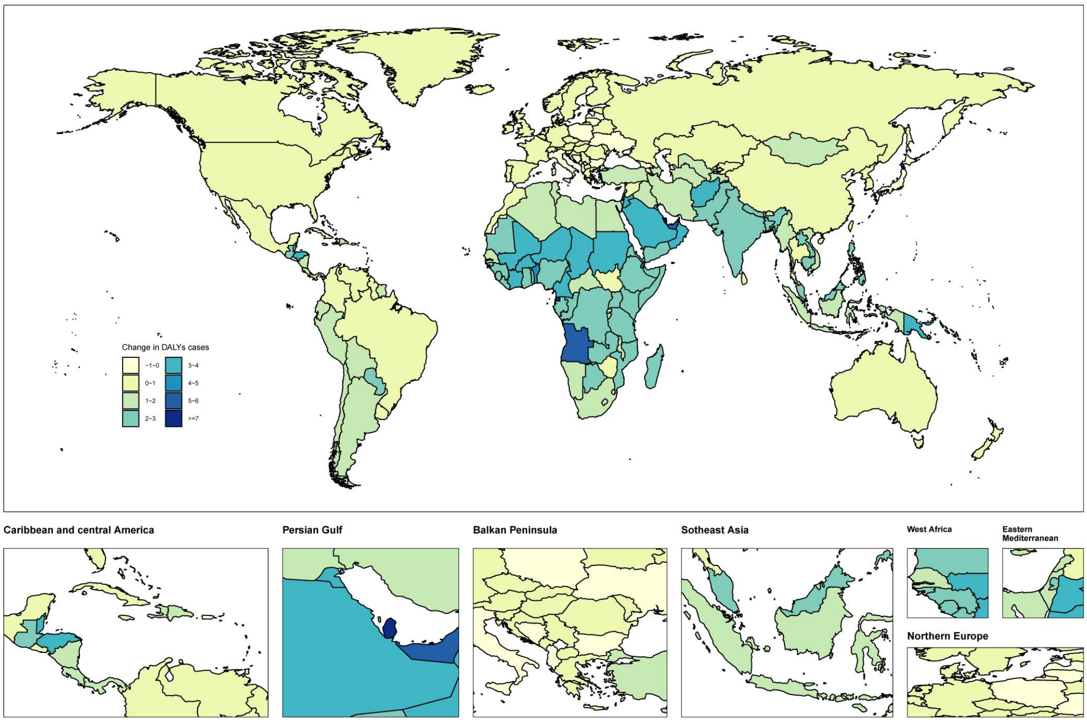

Supplementary Figure 4. Percentage change in DALYs cases across 204 countries in 1990 and 2021.

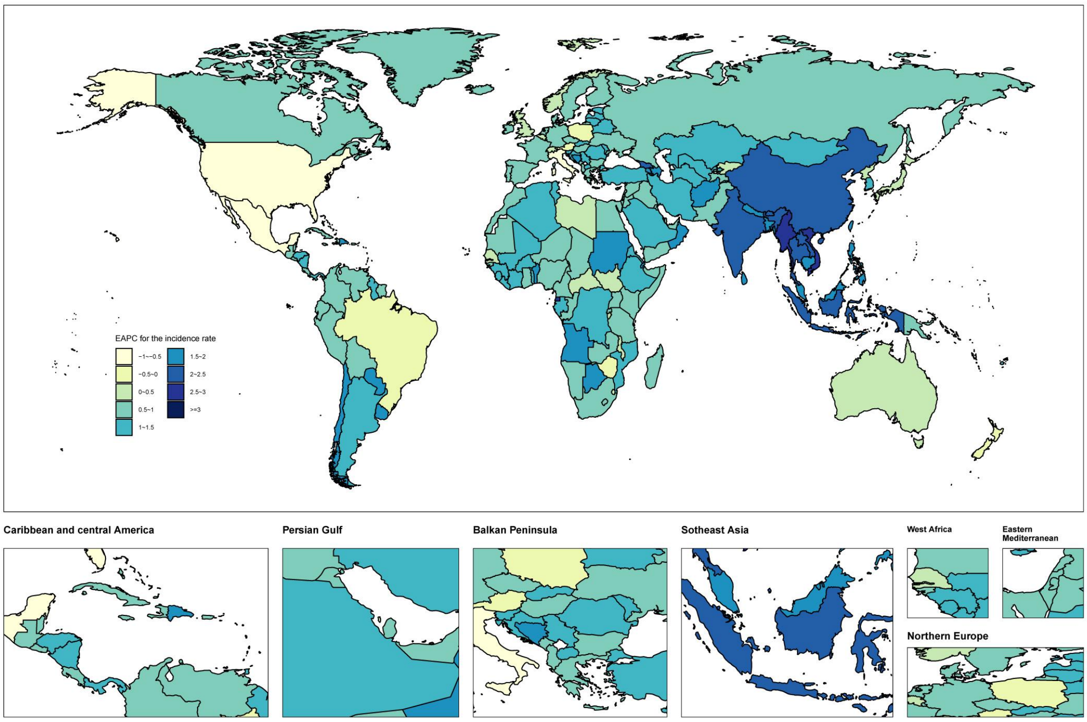

Supplementary Figure 5. EAPC in incidence rates across 204 countries from 1990 to 2021.

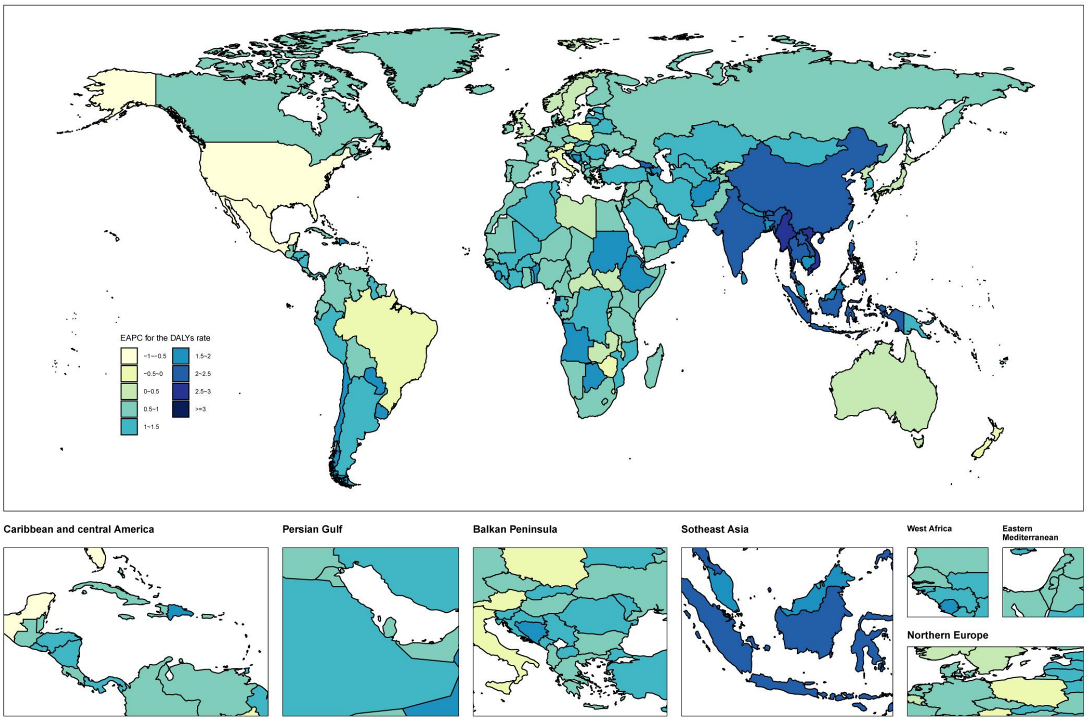

Supplementary Figure 6. EAPC in DALYs rates across 204 countries from 1990 to 2021.

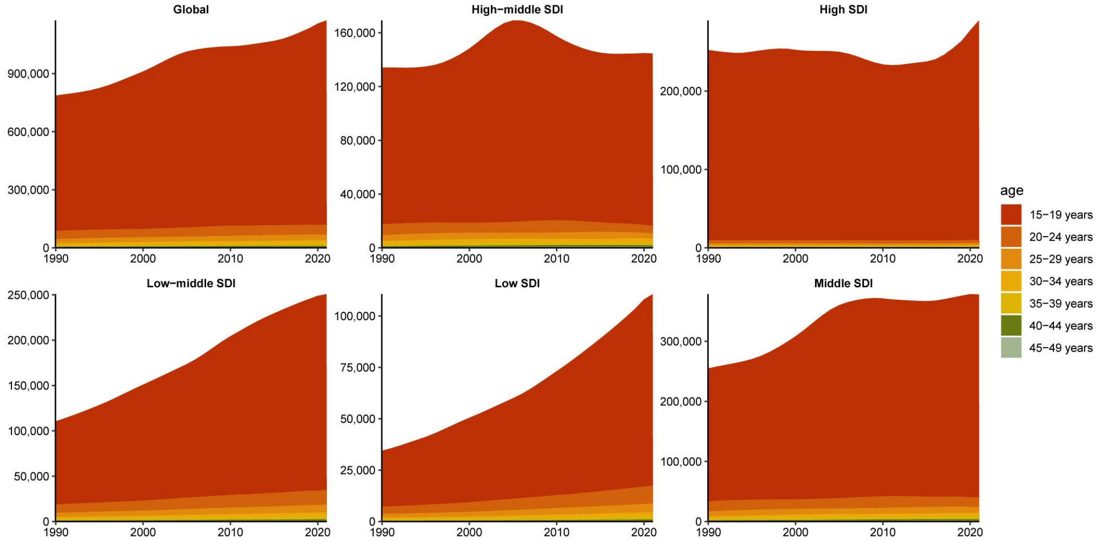

Supplementary Figure 7. Incidence cases of 7 age groups (15-49 years, 5-year intervals) from 1990 to 2021 globally and in 5 territories (low to high SDI).

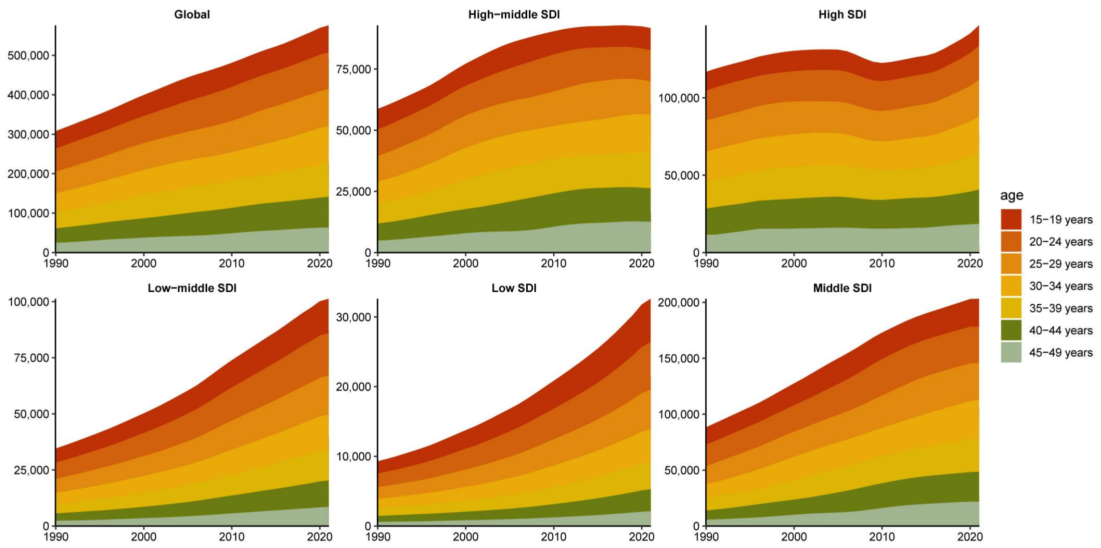

Supplementary Figure 8. DALYs cases of 7 age groups (15-49 years, 5-year intervals) from 1990 to 2021 globally and in 5 territories (low to high SDI).

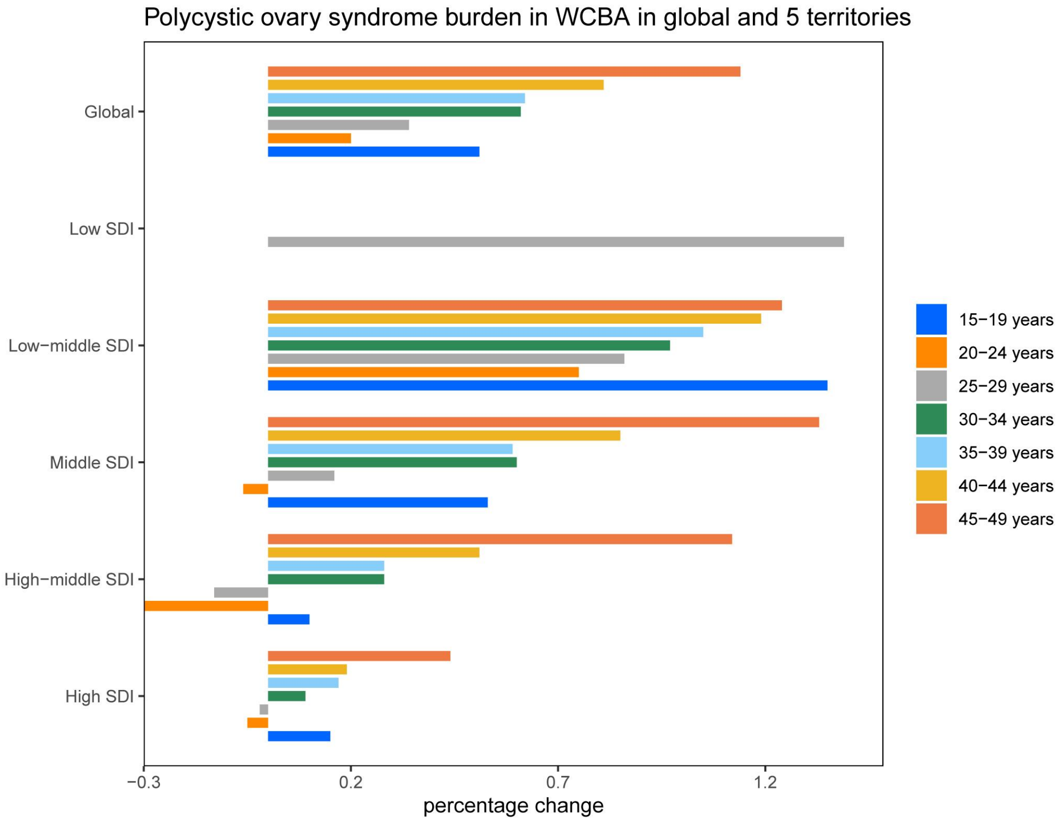

Supplementary Figure 9. Percentage change in incidence cases of 7 age groups globally and in 5 territories in 1990 and 2021.

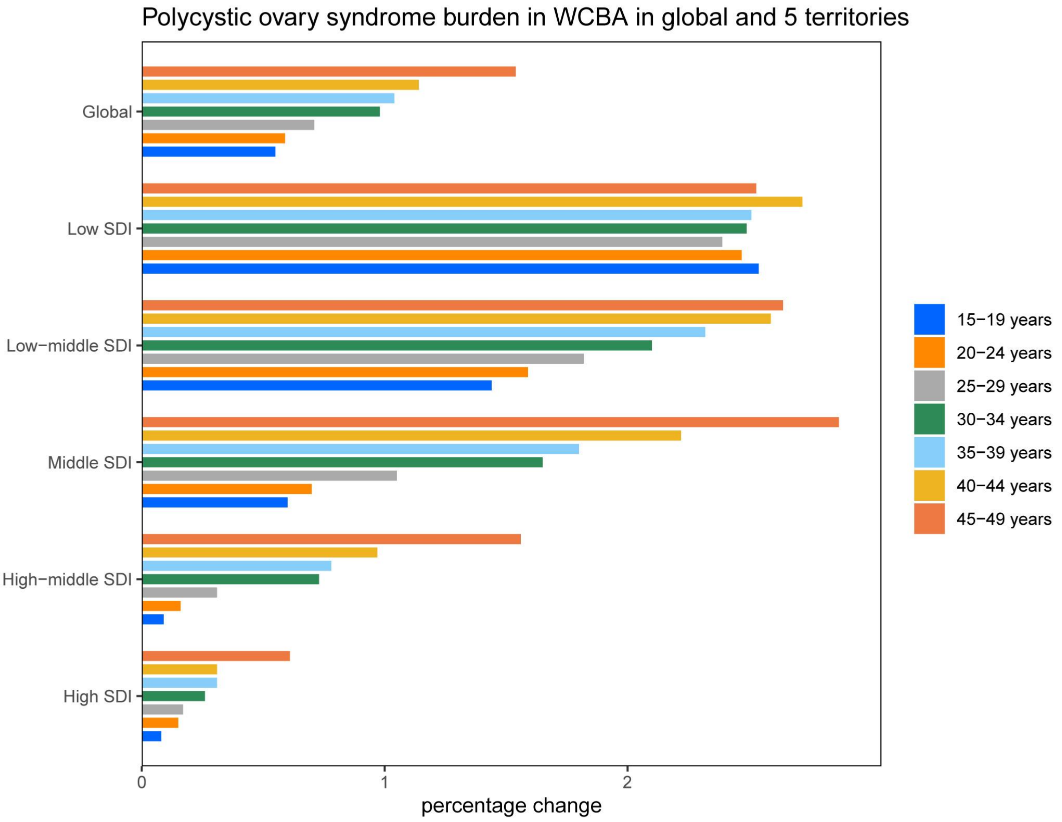

Supplementary Figure 10. Percentage change in DALYs cases of 7 age groups globally and in 5 territories in 1990 and 2021.

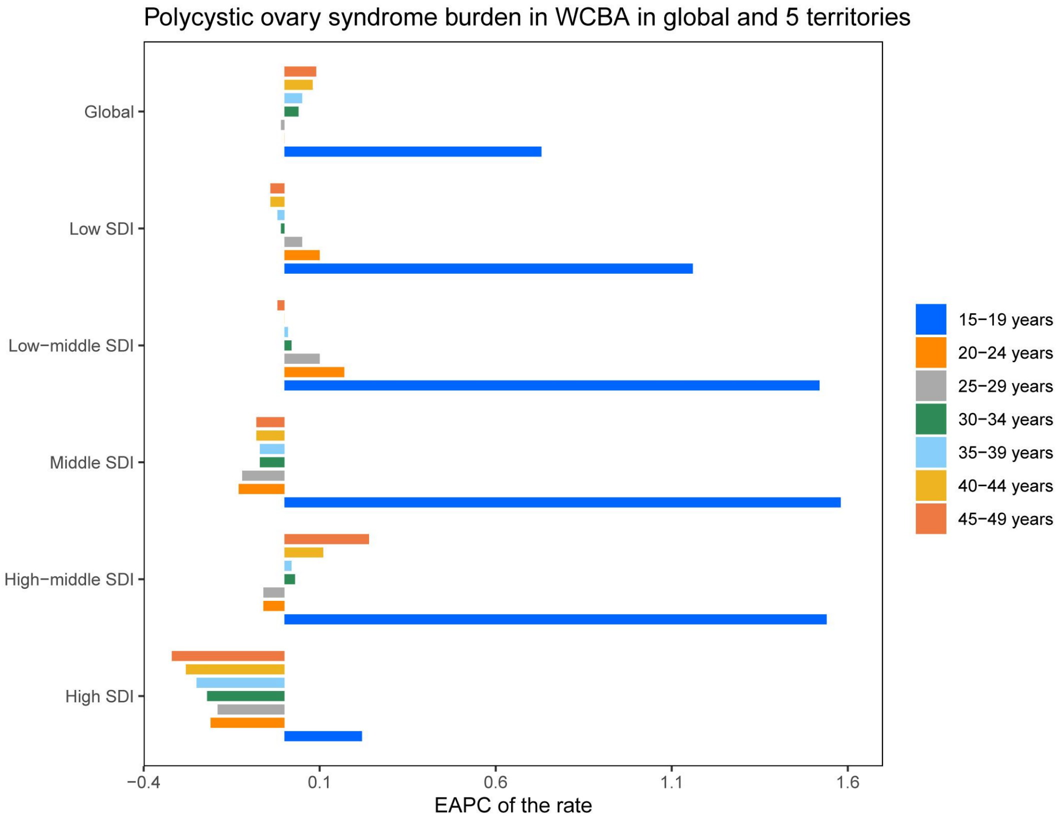

Supplementary Figure 11. EAPC of incidence rates of 7 age groups globally and in 5 territories from 1990 to 2021.

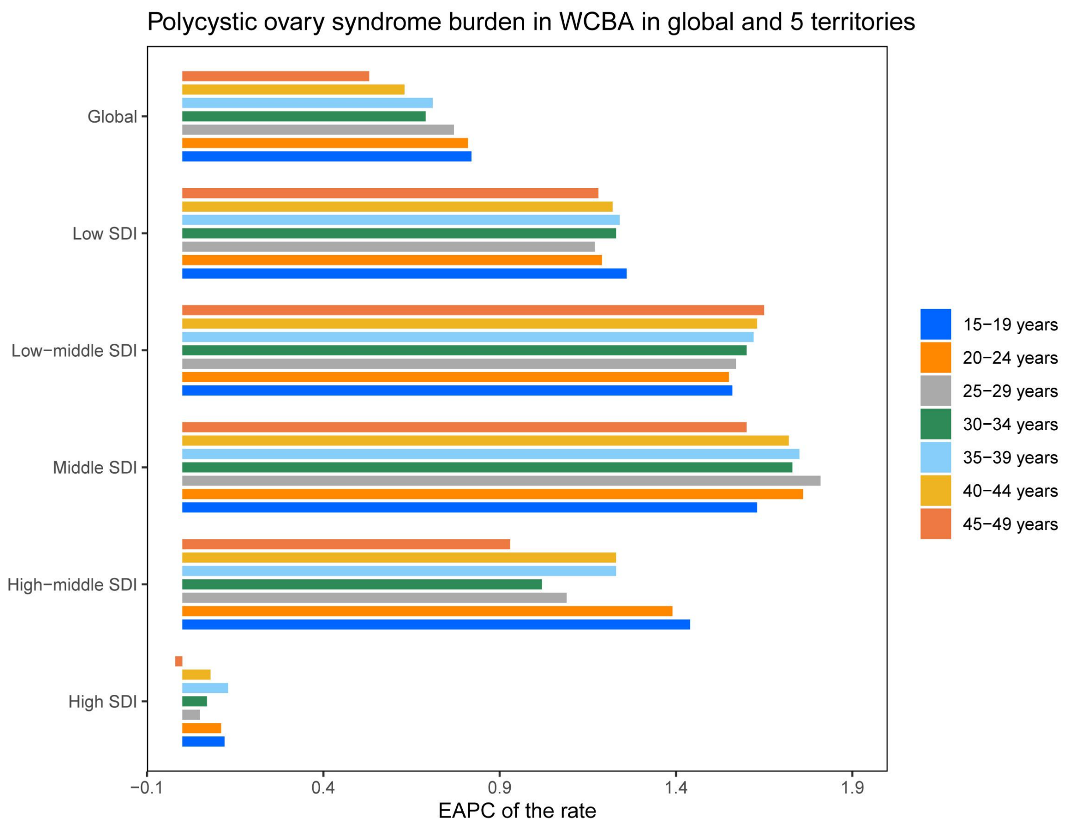

Supplementary Figure 12. EAPC of DALYs rates of 7 age groups globally and in 5 territories from 1990 to 2021.

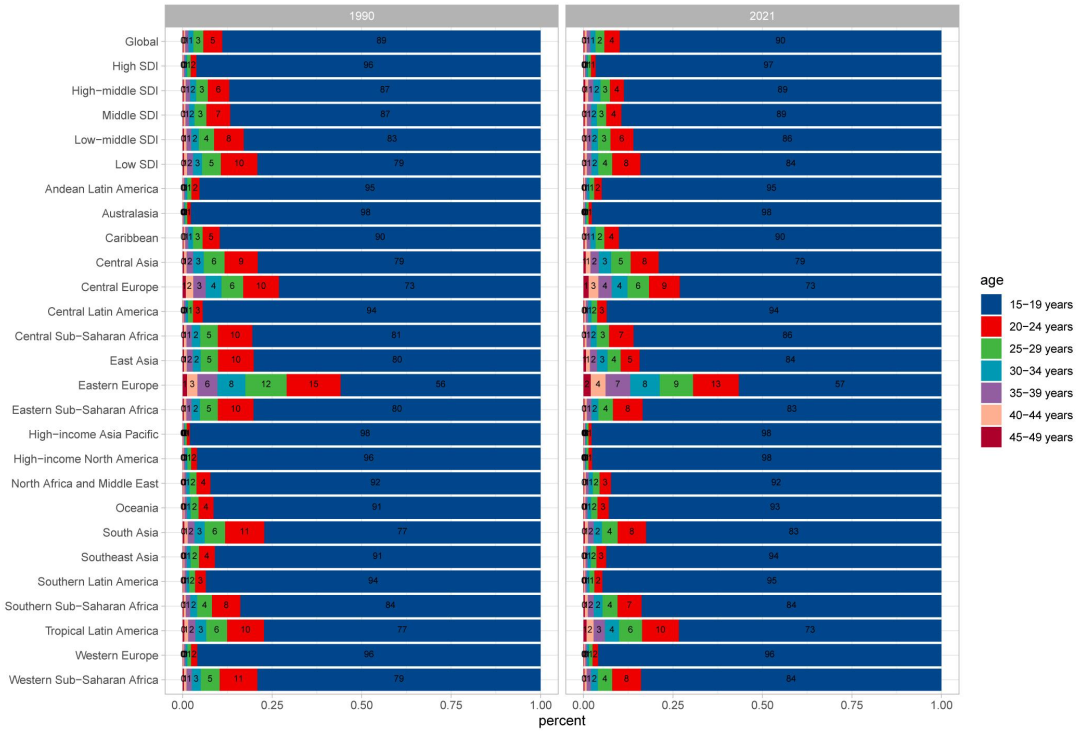

Supplementary Figure 13. The distribution of incidence cases across 7 age groups as percentages globally, in 5 territories, and 21 GBD regions in 1990 and 2021.

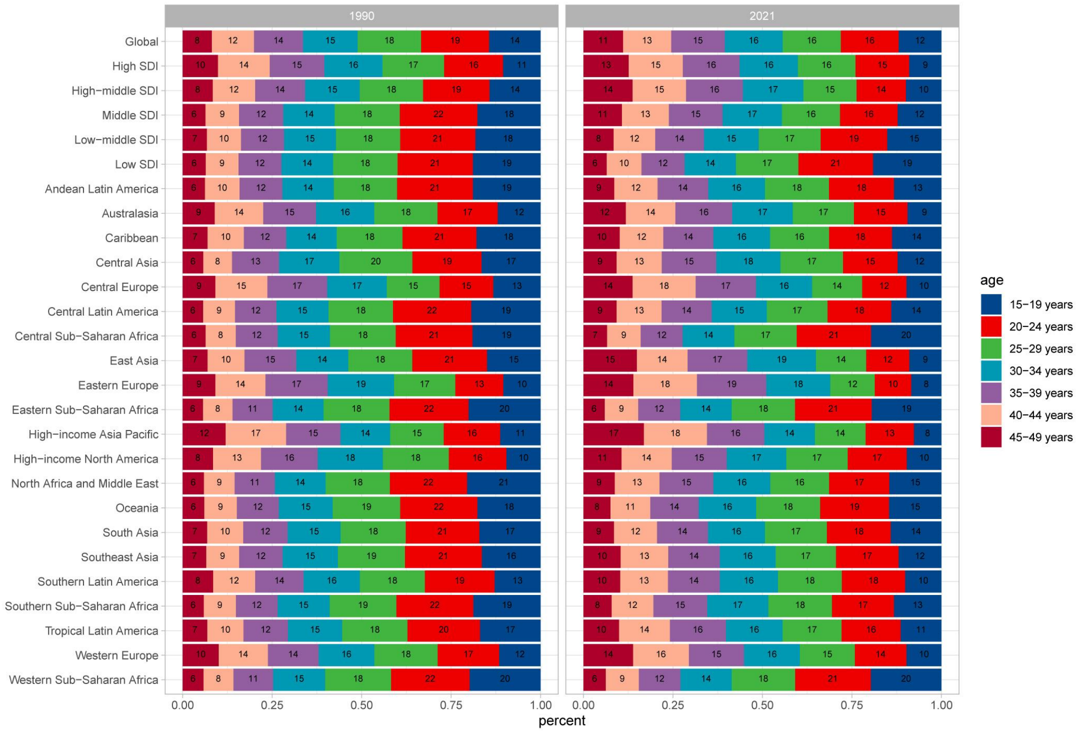

Supplementary Figure 14. The distribution of DALYs cases across 7 age groups as percentages globally, in 5 territories, and 21 GBD regions in 1990 and 2021.

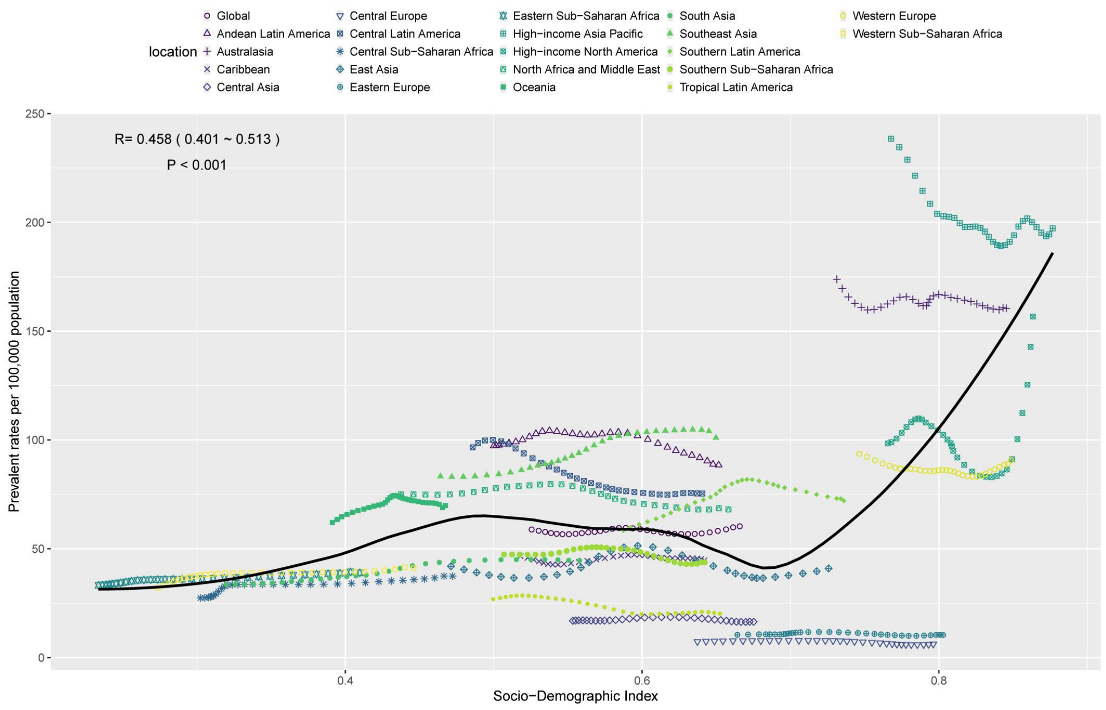

Supplementary Figure 15. The associations between the SDI and incidence rates per 100,000 population of polycystic ovary syndrome in WCBA across 21 GBD regions.

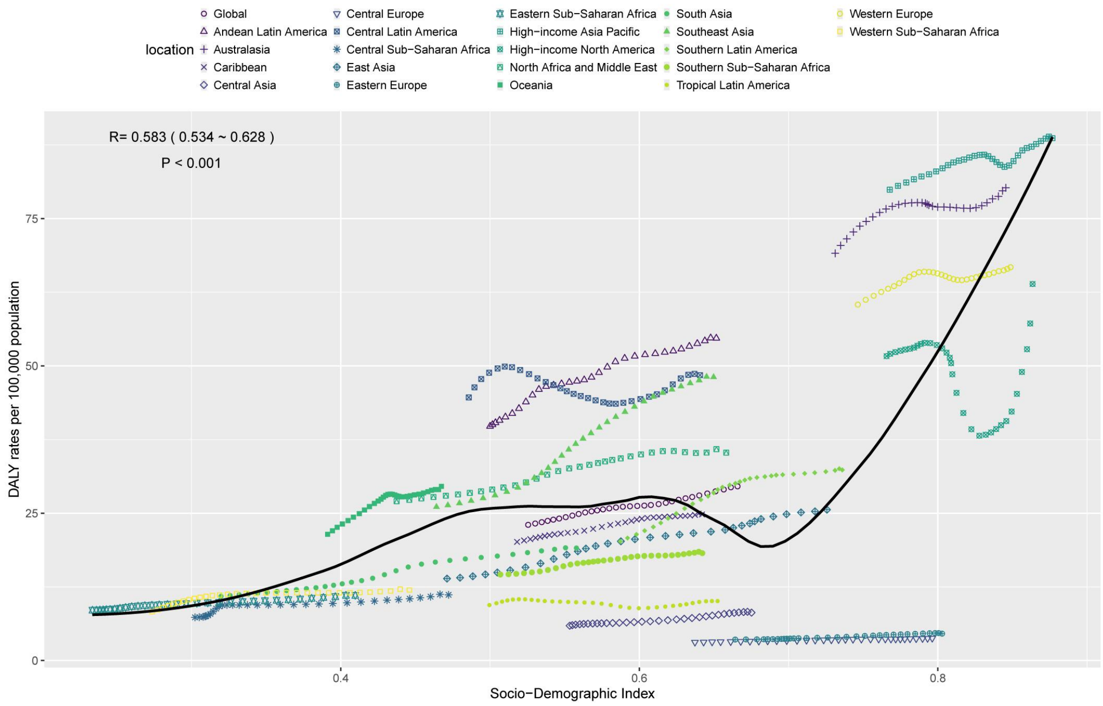

Supplementary Figure 16. The associations between the SDI and DALYs rates per 100,000 population of polycystic ovary syndrome in WCBA across 21 GBD regions.
